# Dizygotic Twin Pregnancy With a Complete Hydatidiform Mole and a Coexisting Viable Fetus

**DOI:** 10.5812/iranjradiol.4488

**Published:** 2011-12-25

**Authors:** Ashraf Moini, Firoozeh Ahmadi, Bita Eslami, Fatemeh Zafarani

**Affiliations:** 1 Department of Endocrinology and Female Infertility, Reproductive Biomedicine Research Center, Royan Institute for Reproductive Biomedicine, ACECR, Tehran, Iran; 2 Department of Obstetrics and Gynecology, Arash Women’s Hospital, Tehran University of Medical Sciences, Tehran, Iran; 3 Department of Reproductive Imaging, Reproductive Biomedicine Research Center, Royan Institute for Reproductive Biomedicine, ACECR, Tehran, Iran; 4 Research Promotion Center, Arash Women’s Hospital, Tehran University of Medical Sciences, Tehran, Iran

**Keywords:** Hydatidiform Mole, Twins, Pregnancy Outcome

## Abstract

Coexistence of a viable fetus with a hydatidiform mole is a rare condition and the diagnosis is very important because of the risk of developing severe complications in pregnancy. The management of these pregnancies is optional, although accurate and great care is required to find early signs of maternal or fetal complications.

Hereby we report a case of dizygotic twin pregnancy with a complete mole and coexisting fetus that resulted in a live neonate.

## 1. Introduction

Coexistence of a viable fetus with a hydatidiform mole is a rare condition with an estimated frequency of 1 in 22,000 to 100,000 pregnancies [1][2]. The diagnosis of twin pregnancy with a complete hydatidiform mole is very important due to the risk of developing severe complications in pregnancy such as early onset of hypertension and pre-eclampsia [3].

In most cases, termination of pregnancy is recommended when the diagnosis is made in early pregnancy [3]. However, assessment of 77 twin pregnancies, comprising a complete hydatidiform mole and a healthy co-twin showed that these pregnancies have a high risk of spontaneous abortion, but about 40% result in livebirth without significantly increasing the risk of persistent gestational trophoblastic disease [4].

Since some patients with this type of pregnancy encounter with some infertility problems, they do not desire to finish their pregnancy. Thus, assessment of more cases was required to establish a standard management. In the present study, we report a case of dizygotic twin pregnancy with a complete mole and coexistent fetus that resulted in a live neonate.

## 2. Case Presentation

**Figure 1:**
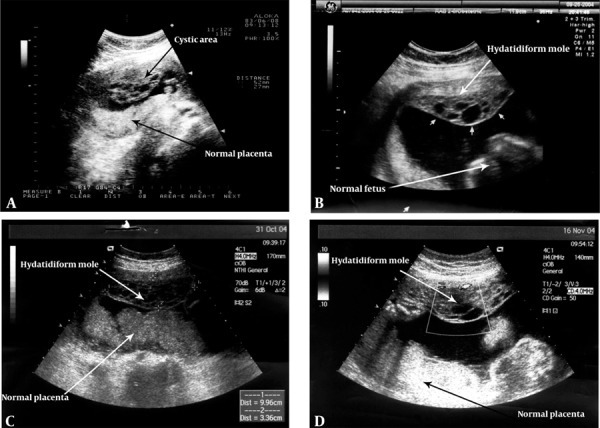
Ultrasonography in a 39-year-old woman with twin pregnancy with a complete hydatidiform mole and a coexisting viable fetus; A, US shows a cystic area at the anterior side of the uterus and normal placenta at 15 weeks of pregnancy; B, Ultrasonography shows a normal fetus with hydatidiform mole at 18 weeks gestation; C, Ultrasonography shows a normal placenta and fetus with hydatidiform mole at 24 weeks gestation; D, Ultrasonography shows placenta and fetus with hydatidiform mole at 28 weeks gestation.

The patient was a 39-year-old woman, gravida 2, para 1 with a normal term male infant delivered by a previous cesarean section 8 years ago. From 2 years ago they had an infertility problem due to azospermia of the second husband. The hormonal profile was normal. The present pregnancy was achieved following ICSI/PESA (intracytoplasmic sperm injection/percutaneous epididymis sperm aspiration). Successive ultrasound examination at 13-week gestation demonstrated a live fetus with a marginal placenta previa. The patient reported spotting from the first weeks of pregnancy until the end of pregnancy. At 15 weeks of pregnancy, a normal placenta and a live fetus was observed. Meanwhile, multiple small vesicles were reported by the sonologist at the anterior side of the uterus which were separated from the normal placenta (Figure 1A). In the next sonographic evaluation (18-20 weeks), a live fetus with a normal placenta was reported again in which a separated area of multiple small vesicles was seen. First, it could be diagnosed as dizygotic twin pregnancy consisting of a normal fetus and a mole (Figure 1B).

At this time, the diagnosis of dizygotic twin molar pregnancy was confirmed and the patient was offered the termination of pregnancy due to future problems. However, she refused to accept and desired to continue her pregnancy. So every week control was recommended.

The next control ultrasound examination showed the normal fetus and the hydatidiform mole clearly (Figure 1C and D).

Successive prenatal examination manifested the patient in good condition without any serious problems for the mother and the fetus.

At 39 weeks gestation, cesarean section was performed because of repeat surgery. A 3150 g normal male infant, with an Apgar score of nine and ten at 1 and 5 minutes, respectively, was delivered. The placenta was extracted completely from the inner uterine wall.

According to the gross examination report, the specimen consisted of a placenta 20 × 15 × 12 cm in diameter and 315 g weight consisting of two parts. One portion with a pink to dark reddish color and spongy consistency resembling a normal placenta and the other part consisted of multiple small vesicles resembling a hydatidiform mole. It seems that the normal placenta and the hydatidiform mole were attached subsequently increasing the gestational age.

Microscopic examination revealed some fragments of the placental tissue. Most of the chorionic villi showed a nearly normal appearance. Foci of villi necrosis and intervillous fibrin deposition were evident. Sections of molar vesicles revealed edematous villi with marked stromal hydropic changes and cistern formation. Foci of trophoblastic proliferation were observed.

Cytogenic analysis of the molar part of the placenta revealed a diploid 46, XX karyotype. After cesarean section, the β-hCG was followed for 1 year finally, returning to the normal level. So, complete remission was diagnosed and the patient has remained clinically well eversince.

## 3. Discussion

There are two different types of pregnancies that present the coexistence of a living fetus and appearance of a molar placenta. One of them is a partial hydatidiform mole and the other is a twin pregnancy with a normal fetus which coexists with a complete or partial hydatidiform mole. In these two separate classifications, the genetic content and both maternal and fetal prognoses are completely different. The incidence of a dizygotic hydatidiform mole with a viable fetus is very rare and this matter is distinguished from a partial molar pregnancy because there are two separate conceptions; namely, a normal placenta linked to the fetus and a molar gestation. In this rare entity, fetuses are chrosomally normal and potentially viable with an increased risk for hemorrhage and medical complications as well as the development of persistent gestational trophoblastic tumor.

In most cases when diagnosis was made in early pregnancy, termination of pregnancy was recommended. The maternal complication and the necessity of termination of pregnancy is an important matter in clinical management. Some studies such as Fishman et al. [4] reported the high frequency (71%) of pregnancy termination because of maternal complications. However, Sebire et al. [2] reported that only 4% of pregnancies were terminated due to maternal complications. Although the data of both studies come through oncologic reports and not exactly through gynecologic and obstetrics reports [5].

In a large study by Vaisbuch et al., they reported 130 cases of twins with CHMF (complete hydatidiform mole and coexistent fetus) pregnancy of which 41% were terminated because of the positive probability of serious maternal complications [6].

On the other hand, women with hydatidiform mole are at risk of preterm delivery (PTD). Some previous studies reported a greater risk of PTD in women who had a twin pregnancy with CHMF (50-60%) compared with a singleton molar pregnancy (15%) [3]. The recent study by Neimann in 2007 revealed that the risk of PTD after a diploid mole with a viable fetus is similar to that after a singleton molar pregnancy and elective early termination of such pregnancy because of the risk of PTD alone should not be recommended [5]. Another study in 2009 which evaluated the registered data of patients from 1999 to 2006 showed the 50% (7 cases in 14) rate of gestational trophoblastic neoplasia (GTN) after CHMF. Six of these patients were treated by single-agent chemotherapy and only one needed multi-agent chemotherapy [7].

Fetal complications such as spontaneous fetal loss before 24 weeks, intrauterine death and severe PTD before 32 weeks were reported. The chance of an alive fetus in these cases has been estimated between 29% and 38% [2][4] and no fetal anomalies have been yet reported.

Recent literature in 2008 reviewed 24 studies that reported 30 cases of CHMF resulting in a live birth documented in detail [8]. Two of the conceptions occurred following ICSI/ET similar to our study. Cesarean section was reported due to fetal or maternal complications in 14 of 30 cases (46.7%). However, in our study cesarean section was performed because of the previous history of cesarean section in this patient.

Therefore, management of molar pregnancy with an alive fetus is optional, although accurate and great care is required to find early signs of maternal or fetal complications and in the presence of a stable pregnancy, normal karyotype and a normal sonogram, it is reasonable to allow the pregnancy to continue.
